# Immunophenotyping and Activation Status of Maternal Lymphocytes to Predict Spontaneous Preterm Birth in Women With Threatened Preterm Labor: A Prospective Observational Study

**DOI:** 10.1111/aji.70015

**Published:** 2024-12-03

**Authors:** Maeva Wendremaire, Tarik Hadi, Tatiana E. Lopez, Julien Guy, Fabrice Neiers, Carmen Garrido, Emmanuel Simon, Zohra Jaffal, Virginie Bernigal, Marc Bardou, Frédéric Lirussi

**Affiliations:** ^1^ INSERM UMR 1231 Centre for Translational and Molecular Medicine Dijon France; ^2^ Université de Bourgogne Dijon France; ^3^ Department of Cardiac Surgery NYU Langone Medical Center New York New York USA; ^4^ Laboratoire d'hématologie biologique Centre Hospitalo‐Universitaire Dijon France; ^5^ Centre Georges François Leclerc Dijon France; ^6^ Service de Gynécologie‐Obstétrique Centre Hospitalo‐Universitaire Dijon France; ^7^ INSERM CIC‐P 1432 Centre Hospitalo‐Universitaire Dijon Dijon France; ^8^ Université de Franche‐Comté Besançon France; ^9^ Plateforme PACE Laboratoire de Pharmacologie‐Toxicologie Centre Hospitalo‐Universitaire Besançon France

**Keywords:** biomarker, immunophenotyping, preterm labor, prospective study, T cells

## Abstract

**Problem:**

Preterm birth (PTB) remains the leading cause of neonatal morbidity and mortality. Identifying women at high risk of spontaneous preterm labor (PTL) is challenging due to limited efficient diagnostic markers. Since human parturition involves inflammatory immune processes, we hypothesized that phenotyping of maternal peripheral lymphocytes might predict PTL. Therefore, we aimed to explore the relationship between maternal lymphocyte subpopulations and labor onset characterized by delivery within 7 days of admission in women hospitalized for PTL between 24 and 34 weeks of gestation.

**Methods of Study:**

Lymphocyte subpopulations were obtained from peripheral blood samples and characterized by flow cytometry: activated and regulatory T cells, natural killer and B cells, and T_H_1/T_H_2/T_H_17 lymphocytes. Data analysis was conducted retrospectively based on the delivery within 7 days of admission.

**Results:**

Among 167 women admitted for PTL, less than 10% delivered within 7 days post‐admission. HLA‐DR expression was significantly increased on CD4^+^CD8^−^, CD4^−^CD8^+^, and CD4^+^CD8^+^ lymphocytes in women who delivered within 7 days. Subset levels below 5% of CD4^+^CD8^−^HLA‐DR^+^ lymphocytes and 20% of CD4^+^CD8^+^HLA‐DR^+^ lymphocytes were associated with no probability of delivering within 7 days.

**Conclusion:**

Our study suggests that combining these two consecutive markers allowed us to identify 57% of women hospitalized for PTL with no probability of delivering within 7 days while retaining patients who delivered within 7 days. If prospectively validated, these markers may be able to identify patients at high risk of PTB and avoid a significant number of unnecessary admissions and healthcare costs.

**Trial Registration:**

ANSM number: 2010‐A00516‐33; ClinicalTrials.gov identifier: NCT01340222

## 1 Introduction

Preterm birth (PTB) still remains the leading cause of neonatal morbidity and mortality [1, 2, 3]. Spontaneous PTB (sPTB) accounts for two‐thirds of PTB cases; the remaining one‐third are medically indicated, due to maternal or fetal complications [4]. An episode of suspected preterm labor (PTL) is a common reason for hospitalization of pregnant women, with a frequency ranging from 9% to 24% [5]. However, roughly 38% of these women will deliver during their hospitalization for threatened labor and one third will deliver at term [5, 6]. The difficulty for healthcare professionals is to categorize women at risk of preterm delivery in order tailor the management or safe discharge hospitalization. Accurate identification of women at high risk of sPTB remains a current clinical issue.

The diagnosis of sPTL is based on the clinical examination, transvaginal ultrasound measurement of cervical length and determination of fetal fibronectin in cervicovaginal fluid [7]. However, the predictive accuracy of these markers is low and suffers from multiple limitations. Fetal fibronectin testing, in patients with threatened PTL, has not shown benefit in preventing PTB or improving perinatal outcomes, but is associated with higher costs [8]. Levine et al. [9] showed that a shorter cervical length was associated with an increased risk of sPTB in symptomatic women, although the positive predictive value (PPV) remains limited. The combination of transvaginal cervical length measurement and fetal fibronectin testing, to identify women at risk, has been evaluated, with conflicting results [10, 11, 12, 13].

Parturition is a multistage process characterized by increased uterine contractility, cervical ripening, and activation of the chorioamniotic membranes [14, 15]. It is now well established that delivery results from the priming of circulating leukocytes [16, 17] and the massive infiltration of immune cells into the myometrium [18, 19, 20]. A role for the adaptive immune system, specifically T cells, in the onset of spontaneous labor has prompted numerous studies, both within the utero‐placental sphere and in the maternal circulation [16, 21–24]. Significant changes in the proportion of circulating CD4^+^ and CD8^+^ T cells were observed in preterm laboring women compared to non‐laboring women [16]. Recently, specific signatures of the immune system, particularly of T lymphocytes, have been associated with labor using single‐cell RNA sequencing [24]. Garcia‐Flores et al. [24] demonstrated that these placenta‐derived signatures associated with labor could be monitored in the maternal circulation, suggesting their potential as non‐invasive markers for predicting sPTB. More precisely, dynamic changes of the maternal immune system between labor induction and the establishment of active labor were investigated through the collection and analysis by single‐cell mass cytometry of blood samples at five different timepoints during labor progression [22]. Notably, the transition to active labor was marked by immune activation of pro‐inflammatory signaling responses.

Regulatory T (Treg) cells increase systemically and locally during human pregnancy [25, 26], but decrease dramatically during labor, either at term or preterm. Treg cell number and suppressive activity are further reduced in women undergoing PTL [27, 28, 29]. Besides, systemic immune activation is also triggered by pregnancy, with cellular adaptive immune components, T cells and B cells, undergoing specific changes. T cells show increased basal activation and proliferation, while B cells exhibit more memory‐like and activated phenotypes [30].

Given that labor‐associated specific immune signatures could be tracked in the maternal circulation, we hypothesized that phenotyping of maternal peripheral immune cells might help to predict the reality of PTL. Therefore, we evaluated the association between lymphocyte subpopulations and delivery in women hospitalized for PTL between 24 and 34 weeks of gestation (WOG). The secondary objective was to assess whether lymphocyte subpopulations could predict delivery within the first 7 days after admission.

## 2 Materials and Methods

### 2.1 Ethics Approval, Study Design, and Patient Recruitment

This study was approved by the French Medicines Agency and the local ethics committee (CPP‐Est, Dijon, France). Written informed consent was obtained from all patients. The study was designed as a prospective observational study. Inclusion criteria were as follows: Threatened PTL occurring between 24 and 34 WOG, defined according to validated criteria, that is, by (1) the presence of regular uterine contractions, lasting at least 30 s with a frequency of ≥4 contractions every 30 min and confirmed by an external tocogram; (2) a cervical dilatation of 3 cm or more, to focus on true PTL, in nulliparous women and 1–3 cm in primiparous or multiparous women; and (3) a cervical obliteration >50%, or a cervix less than 25 mm according to the echography, if this information is available. Non‐inclusion criteria were as follows: woman with a spontaneous rupture of membranes with confirmed or probable chorioamnionitis, inflammatory or autoimmune disease, suspected or confirmed infectious disease, including HIV, HCV, HBV, anti‐inflammatory drug, or immunomodulator treatment, patient less than 18 years old, patient not affiliated to social security.

We enrolled 200 women in two neighboring level 3 maternity units, Dijon and Besançon. Two 5 mL blood samples were taken on admission for threatened PTL. All clinical and biological data were collected prospectively and reported in the case report form. There was no secondary exclusion, even in the case of early discharge if the threat of premature birth disappeared.

### 2.2 Immune Cells Subsets Staining

Lymphocytes subpopulations were characterized with a mixture of fluorophore‐coupled monoclonal antibodies purchased from BD Biosciences and reported in Table S1. One hundred microliters of antibody mix were added to the blood sample and incubated 20 min at 4°C. After incubation, a cold 1X Red Blood Lysis Buffer was added, and lysis was performed for 10 min at room temperature. After two washes with Phosphate Buffered Saline (PBS) and centrifugation, pellets were resuspended in 400 µL PBS before fluorescence‐activated cell sorting (FACS) cytometry. For T_H_1/T_H_2/T_H_17 lymphocytes characterization, peripheral mononuclear cells (PBMC) were isolated using gradient centrifugation (400 × *g* without break for 20 min, at room temperature) with Ficoll‐Paque Plus and subsequently stimulated for 4 h in presence or absence of PMA/ionomycin. Finally, fixed/permeabilized cells were stained with T_H_1/T_H_2/T_H_17 antibody cocktail according to the manufacturer's protocol (Table S1). After two washes with PBS and centrifugation, pellets were resuspended in 400 µL PBS before FACS analysis.

### 2.3 Cytometric Analyses of Blood Immune Cells Subsets

Flow Cytometry experiments were performed at the ImaFlow core facility (US58 BioSanD, Dijon, France). Cytometric analysis was performed on an LSRII cytometer (Becton Dickinson, Pont‐de‐Claix, France) using FACS Diva 6.1.2 software (Becton Dickinson, Pont‐de‐Claix, France). Cells were acquired in a forward scatter/side scatter (FSC/SSC) dot plot. Lymphocytes were gated in a CD45/SSC dot plot and staining was analyzed within this primary gate according to different gating strategies (Figures S1 and S2). The different lymphocytes subpopulations were identified as follows: CD4^+^ T cells (CD45^+^CD3^+^CD4^+^CD8^−^), CD8^+^ T cells (CD45^+^CD3^+^CD4^−^CD8^+^), Dual Positive cells (CD45^+^CD3^+^CD4^+^CD8^+^), and Gamma Delta cells (CD45^+^CD3^+^CD4^−^CD8^−^). The proportions of each of these subpopulations were expressed as a percentage of CD3^+^ lymphocytes. Each HLA‐DR^+^ cells population was analyzed within its respective parent gate: CD4^+^CD8^−^ gate for CD4^+^CD8^−^HLA‐DR^+^ cells, CD4^−^CD8^+^ gate for CD4^−^CD8^+^HLA‐DR^+^ cells, and CD4^+^CD8^+^ gate for CD4^+^CD8^+^HLA‐DR^+^ cells. Regulatory CD4^+^ and CD8^+^ T cells were identified as CD45^+^CD3^+^CD4^+^CD8^−^CD25^hi^CD127^lo^ and CD45^+^CD3^+^CD4^−^CD8^+^CD25^hi^CD127^lo^ cells, respectively and expressed as a percentage of CD4^+^ and CD8^+^ T lymphocytes, respectively. The other lymphocytes subpopulations were identified as NK cells (CD45^+^CD3^−^CD16^+^CD56^+^), NKT cells (CD45^+^CD3^+^CD16^+^CD56^+^), and B cells (CD45^+^CD3^−^CD19^+^). The proportions of each subpopulation were expressed as percentage of total lymphocytes and analyses were performed by acquiring 10 000 events in the lymphocytes stopping gate. For T_H_1/T_H_2/T_H_17 lymphocytes analysis, cells were gated in a FSC/SSC dot plot and staining was analyzed within this primary gate to discriminate first CD4^+^ cells, and then T_H_1 (IFN‐γ^+^), T_H_2 (IL‐4^+^), and T_H_17 (IL‐17^+^) subsets (Figure S3). The proportions of each subpopulation were expressed as a percentage of CD4^+^ lymphocytes and analyses were performed by acquiring 100,000 events in the lymphocytes stopping gate.

To determine the cut‐off point between background fluorescence and positive populations, we used fluorescence minus‐one (FMO) and isotype controls for each panel [31]. For our multicolor flow cytometry experiments, compensation was performed using VersaComp Antibody Capture Beads (B22804, Beckman Coulter, Villepinte, France) to correct spectral overlap between fluorochromes. Last, to compare the median fluorescence intensity (MFI) between patients whose staining were performed on different days, application settings were created and applied to each new experiment. Cytometer setup & tracking beads (642412, BD Biosciences, Pont‐de‐Claix, France) were run daily.

### 2.4 Statistical Analysis

We followed a pre‐specified statistical analysis plan. Results were expressed as the percentage (± standard error to the mean, SEM) of each subpopulation among its specific lymphocytes population and as MFI for HLA‐DR^+^ cells in arbitrary units. Several analyses were performed according to our study objectives. First, we compared the different lymphocyte subsets in patients who delivered or did not deliver within the first 7 days after admission for threatened PTL. A *p* value < 0.05 was considered significant, no formal correction for multiple testing was conducted. We describe the ability of lymphocytes activation status to discriminate between women who delivered or not within 7 days post admission as area under the receiver operating characteristic (ROC) curve (AUC) sensitivity, specificity, positive and negative predictive values [32]. If the lower limit of the 95% CI of the AUC is > 0.5, we consider the measurement to have discriminative potential. A two‐level analysis of the receiver operating characteristic curves and the areas under the curve was conducted in significant variables associated with delivery, to determine two consecutive cut‐off levels to select all the patients who delivered within the first 7 days post admission (sensitivity of 100%).

## 3 Results

### 3.1 Cohort Characteristics

During the study period, 200 patients were enrolled (139 in Dijon and 61 in Besançon). Four patients withdrew their consent, and 29 patients were excluded because their cytometric data could not be used. The demographic and obstetric characteristics of the 167 study participants are shown in Table 1. Of the 167 patients admitted with threatened PTL, only 16 delivered within the first 7 days after admission (10 [7.2%] at Dijon and 6 [9.8%] at Besançon); the mean gestational age of these patients was 29.3 ± 3.3 WOG. Overall, the majority of women (59.3%) were nulliparous, but no differences were observed between those who delivered within 7 days (43.8%) and those who did not (60.9%). Gestational age at admission was 29.2 (±2.6) WOG and 25 women (15%) had a history of PTL.

**TABLE 1:** Maternal demographic and obstetrical characteristics at the time of admission.

Characteristics	Total	Delivery >7 days	Delivery ≤7 days	*p* value^a^
Number of women	167	151	16	
Age, years	28.6 ± 5.2	28.7 ± 5.2	27.6 ± 5.6	0.29
BMI, kg/m^2^	22.0 ± 3.4	21.9 ± 3.4	23.0 ± 3.6	0.28
<25	129 (83.2)	117 (84.2)	12 (75)	
25–29.9	19 (12.3)	16 (11.5)	3 (18.8)	
≥30	7 (4.5)	6 (4.3)	1 (6.3)	
Tobacco use				
Never	79 (68.7)	70 (69.3)	9 (64.3)	
Remote	11 (9.6)	10 (9.9)	1 (7.1)	
During pregnancy	25 (21.7)	21 (21.9)	4 (28.6)	
Parity	0.64 ± 0.95	0.65 ± 0.99	0.56 ± 0.51	0.59
0	99 (59.3)	92 (60.9)	7 (43.8)	
1–2	58 (34.7)	49 (32.5)	9 (56.3)	
≥3	10 (6)	10 (6.6)	0 (0)	
Gestity	2.06 ± 1.61	2.02 ± 1.57	2.47 ± 1.96	0.41
0	6 (3.6)	5 (3.3)	1 (6.7)	
1–2	114 (68.7)	105 (69.5)	9 (60)	
≥3	46 (27.7)	41 (27.2)	5 (33.3)	
Preterm labor history	25 (15.0)	22 (14.6)	3 (18.8)	0.71
First/second trimester vaginal bleeding	18 (17.1)	17 (18.1)	1 (9.1)	0.69
Gestational age at admission, WOG	29.2 ± 2.6	29.3 ± 2.5	28.4 ± 3.4	0.35
Gestational age at delivery, WOG	36.6 ± 4.3	38.0 ± 2.8	29.3 ± 3.3	**<**0.001
Uterine contractions at admission	87 (82.1)	78 (80.4)	9 (100)	0.36
Delay to care >12 h	17 (13.3)	14 (12.0)	3 (27.3)	0.16
Admission temperature, °C	37.0 ± 0.3	37.0 ± 0.3	37.1 ± 0.3	0.43
CRP, mg/L	7.3 ± 11.9	6.7 ± 10.3	13.1 ± 22.3	0.07
Amniotic fluid index, cm				
<5	0	0	0	
5–8	6 (3.6)	6 (4.0)	0	
≥8	142 (85.0)	131 (86.8)	11 (68.8)	
Vaginal bacterial infection	66 (46.8)	60 (46.5)	6 (50)	>0.99
Positive urine cytobacteriological examination	23 (15.5)	21 (15.4)	2 (16.7)	>0.99

*Note:* Results are given as numbers and percentages for categorical variables, or as means and standard deviations for continuous variables.

Abbreviations: BMI, body mass index; CRP, C‐reactive protein; WOG, weeks of gestation.

^a^ *p* values estimated using *t* test or Fisher's exact test to compare the two groups.

### 3.2 Lymphocyte Phenotyping

**FIGURE 1:**
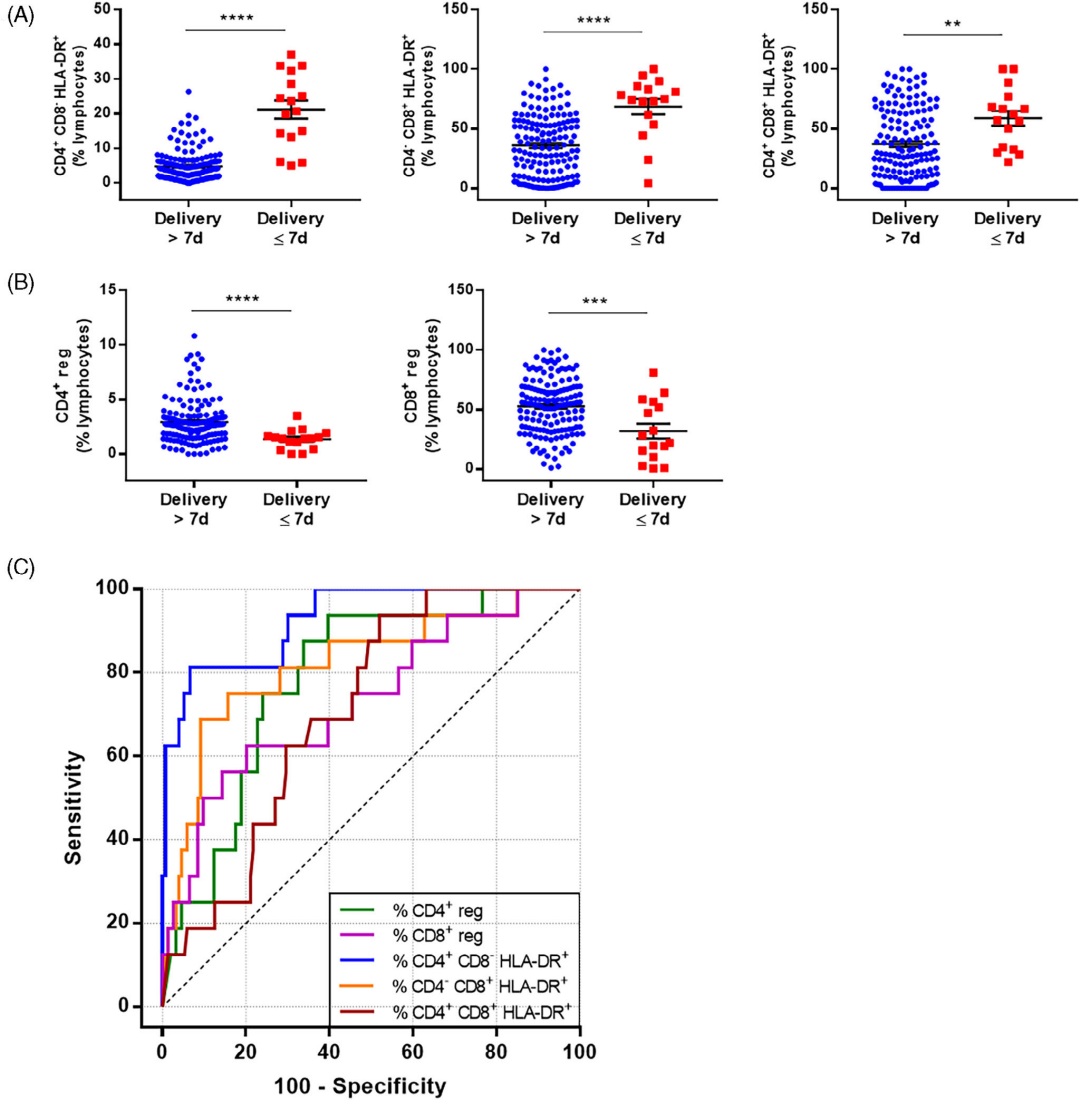
CD4^+^ and CD8^+^ lymphocyte populations and activation status according to delivery or not within 7 days post admission. Percentages of lymphocytes are represented as scatter dot plot. (A) Percentage of CD4^+^CD8^−^HLA‐DR^+^, CD4^−^CD8^+^HLA‐DR^+^, CD4^+^CD8^+^HLA‐DR^+^ lymphocytes. (B) Percentage of CD4^+^ and CD8^+^ regulatory lymphocytes. **p < 0.01; ***p < 0.001; ****p < 0.0001. (C) ROC curve of the percentage of CD4^+^CD8^−^HLA‐DR^+^, CD4^−^CD8^+^HLA‐DR^+^, CD4^+^CD8^+^HLA‐DR^+^, CD4^+^ and CD8^+^ regulatory lymphocytes in predicting delivery within 7 days post admission.

We included many immunological biomarkers, in our analysis, in order to obtain a global immunological “signature” rather than to isolate a specific marker. All biological parameters examined are summarized in Table S2. We found no difference in the proportion of CD4^+^CD8^−^, CD4^−^CD8^+^, and CD4^+^CD8^+^ lymphocytes between women who gave birth within 7 days of admission and those who did not (Figure S4A‐E), whereas their activation status was dramatically modified. Indeed, CD4^+^CD8^−^HLA‐DR^+^, CD4^−^CD8^+^HLA‐DR^+^, and CD4^+^CD8^+^HLA‐DR^+^ lymphocytes percentages were significantly higher in women with delivery ≤7 days than in women with delivery >7 days (21.2 ± 2.6 vs. 4.8 ± 0.4, 68.5 ± 6.5 vs. 36.0 ± 2.1, 58.9 ± 6.2 vs. 37.2 ± 2.4 for CD4^+^CD8^−^HLA‐DR^+^, CD4^−^CD8^+^HLA‐DR^+^, and CD4^+^CD8^+^HLA‐DR^+^ lymphocytes, respectively) (Figure 1A, Table S2). This difference in HLA‐DR expression was significant for the number of positive cells but not for the MFI (Figure S4B‐F). In contrast, CD4^+^ reg and CD8^+^ reg lymphocyte subpopulations were significantly lower in women who delivered within 7 days of admission and those who did not (1.4% ± 0.2% vs. 2.9% ± 0.2%, 32.0% ± 6.3% vs. 52.8% ± 1.9%, for CD4^+^ reg and CD8^+^ reg lymphocytes, respectively) (Figure 1B, Table S2). All other lymphocyte subpopulations such as Gamma Delta, NK, NKT, B (Figure S5) and T_H_1/T_H_2/T_H_17 (Figure S6) showed no significant difference between patients with delivery ≤7 days and those with delivery >7 days (Table S2).

We then evaluated the diagnostic accuracy of these five parameters using ROC curve analysis (Figure 1C). To retain all the patients who gave birth within 7 days, we set the sensitivity of the markers at 100%, corresponding to a negative predictive value (NPV) of 100%. For the prediction of delivery ≤7 days, the highest AUC and specificity was observed for CD4^+^CD8^−^HLA‐DR^+^, with 0.93% (0.87%–0.99%) and 63.4% (55.2%–71.0%), respectively (Table 2). The cut‐off was then set at 5% of CD4^+^CD8^−^HLA‐DR^+^ lymphocytes of the CD4^+^CD8^−^ population. Unfortunately, the corresponding PPV was relatively low. In fact, only 22.9% of the patients above the cut‐off value actually gave birth within the first 7 days after admission for threatened PTL.

**TABLE 2:** Diagnostic accuracy of lymphocytes populations and activation status in predicting delivery within 7 days post admission (primary analysis).

Lymphocyte population	AUC	95% CI	Cut‐off	Sensitivity, %	Specificity, %	PPV, %	NPV, %
CD4^+^CD8^‐^HLA‐DR^+^	0.93	0.87–0.99	≥5.0	100 (79.4–100)	63.4 (55.2–71.0)	22.9	100
CD4^‐^CD8^+^HLA‐DR^+^	0.82	0.70–0.94	≥4.0	100 (79.4–100)	15.0 (9.8–21.7)	11.0	100
CD4^+^CD8^+^HLA‐DR^+^	0.71	0.61–0.82	≥21.4	100 (79.4–100)	36.8 (29.2–45.0)	14.3	100
CD4^+^ reg	0.79	0.69–0.89	<3.5	100 (79.4–100)	23.4 (16.9–30.9)	12.0	100
CD8^+^ reg	0.73	0.60–0.87	<81.1	100 (79.4–100)	14.9 (9.7–21.6)	10.9	100

Abbreviations: AUC, area under curve, CI, confidence interval; NPV, negative predictive value; PPV, positive predictive value.

**FIGURE 2:**
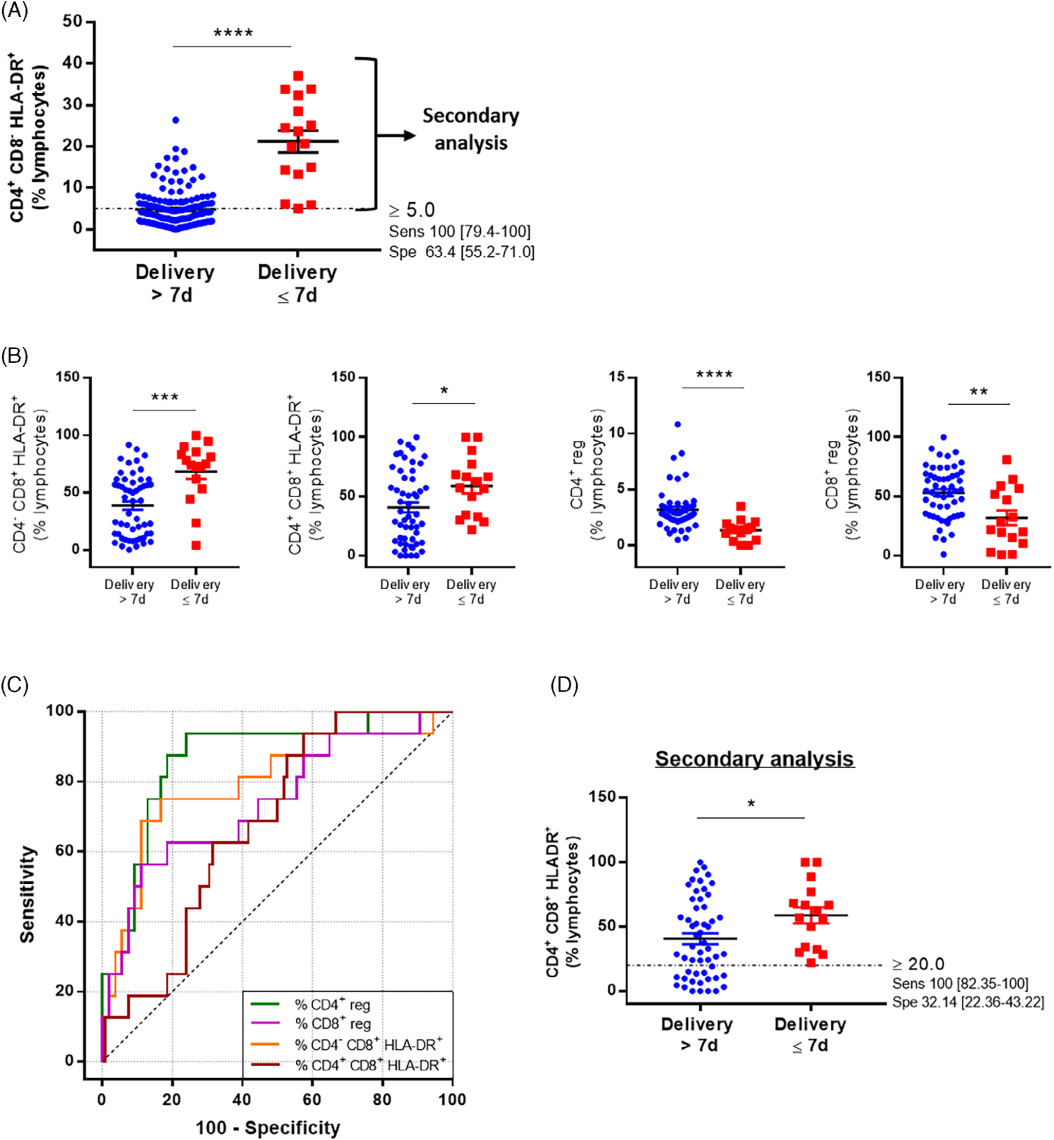
Secondary analysis. (A) Selection of patients for whose percentage of CD4^+^CD8^−^HLA‐DR^+^ lymphocytes is above the 5% threshold for the secondary analysis. (B) Percentage of CD4^−^CD8^+^HLA‐DR^+^, CD4^+^CD8^+^HLA‐DR^+^, CD4^+^ and CD8^+^ regulatory lymphocytes according to delivery or not within 7 days post admission. *p < 0.05; **p < 0.01; ***p < 0.001; ****p < 0.0001. (C) ROC curve of the percentage of CD4^−^CD8^+^HLA‐DR^+^, CD4^+^CD8^+^HLA‐DR^+^, CD4^+^ and CD8^+^ regulatory lymphocytes in predicting delivery within 7 days post admission, after selection of patients for whose percentage of CD4^+^CD8^−^HLA‐DR^+^ lymphocytes is above the 5% threshold. (D) Percentage of CD4^+^CD8^+^HLA‐DR^+^ cells with respect to the 20% threshold according to delivery or not within 7 days post admission.

To refine our analysis, we performed a secondary analysis of patients above the threshold (Figure 2A), using the following markers: CD4^−^CD8^+^HLA‐DR^+^, CD4^+^CD8^+^HLA‐DR^+^, CD4^+^ reg, and CD8^+^ reg subpopulations. As shown in Figure 2B, all these markers were significantly different between women with delivery ≤7 days and women with delivery >7 days. Still with a sensitivity of 100%, the ROC curve analysis allowed us to select the percentage of CD4^+^CD8^+^HLA‐DR^+^ lymphocytes as a marker of delivery ≤7 days (Figure 2C). Although its AUC is the lowest among the studied criteria, this indicator has the highest specificity of 33.3% (21.1–47.5) and a PPV of 28.6% (Table 3). For this parameter, a cut‐off of 20% was determined below which none of the patients of our study delivered within 7 days post‐admission (Figure 2D).

**TABLE 3:** Diagnostic accuracy of lymphocyte populations and activation status to predict delivery within 7 days of admission after selection of patients for whose percentage of CD4^+^CD8^‐^HLA‐DR^+^ lymphocytes was above the 5% threshold.

Lymphocyte population	AUC	95% CI	Cut‐off	Sensitivity, %	Specificity, %	PPV, %	NPV, %
CD4^‐^CD8^+^HLA‐DR^+^	0.80	0.66–0.94	≥3.9	100 (79.4–100)	5.6 (1.2–15.4)	21.9	100
CD4^+^CD8^+^HLA‐DR^+^	0.68	0.55–0.81	≥20.6	100 (79.4–100)	33.3 (21.1–47.5)	28.6	100
CD4^+^ reg	0.87	0.76–0.97	<3.5	100 (79.4–100)	24.1 (13.5–37.6)	25.4	100
CD8^+^ reg	0.74	0.59–0.89	<82.5	100 (79.4–100)	9.3 (3.1–20.3)	22.2	100

Abbreviations: AUC, area under curve; CI, confidence interval; NPV, negative predictive value; PPV, positive predictive value.

## 4 Discussion

**FIGURE 3:**
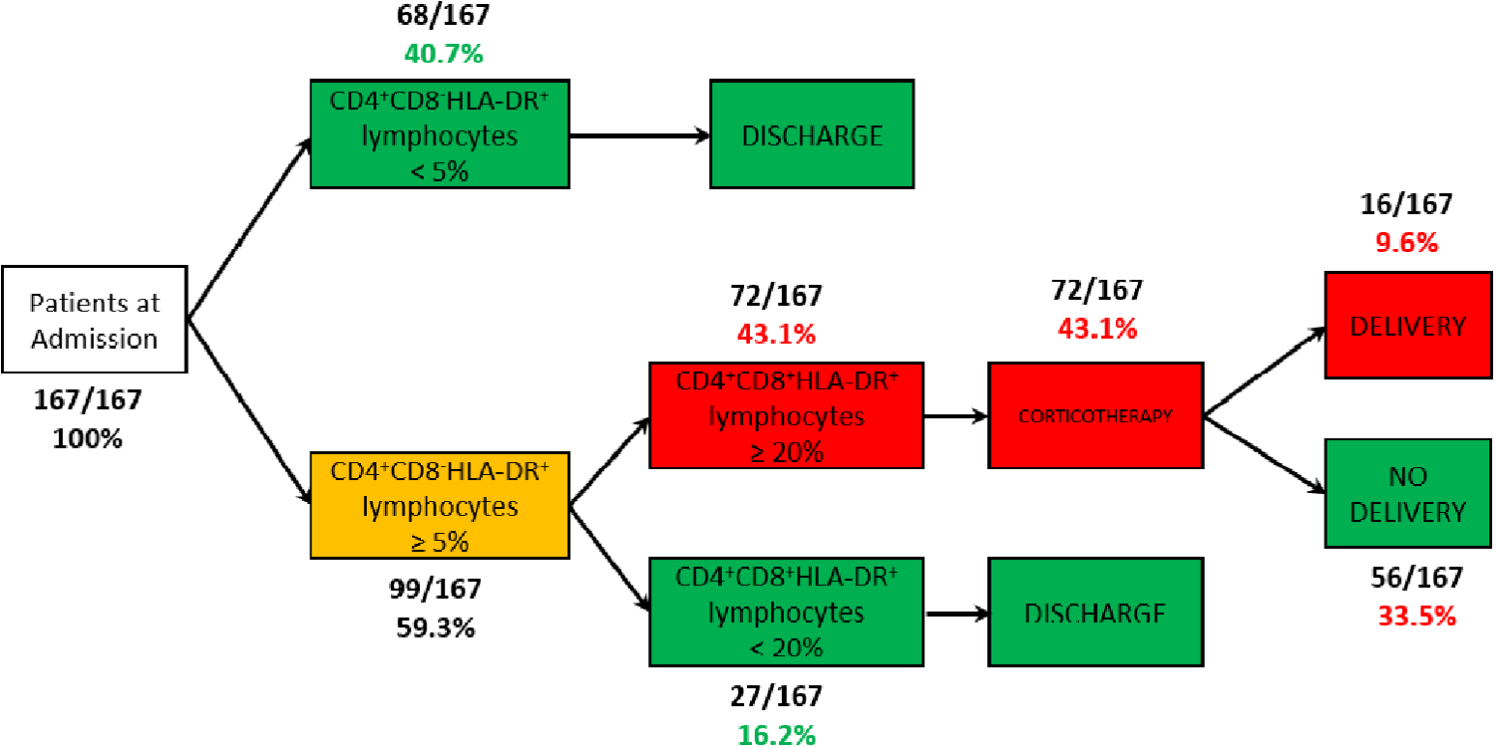
Flowchart of study patients according to their lymphocyte immunophenotyping results at admission, combining the percentages of CD4^+^CD8^−^HLA‐DR^+^ and CD4^+^CD8^+^HLA‐DR^+^ lymphocytes.

In this study, we confirmed that among women admitted for threatened PTL, only a few of them (9.6%) gave birth within 7 days. There was no apparent difference in the proportion of CD4^+^CD8^−^, CD4^−^CD8^+^, and CD4^+^CD8^+^ lymphocytes between women who delivered within 7 days of admission and those who did not. However, the activation status increased dramatically in women who gave birth within 7 days compared to those who did not. On the contrary, CD4^+^ reg and CD8^+^ reg lymphocyte subpopulations were significantly lower in women who gave birth within 7 days compared with those who did not. All patients who delivered within 7 days of admission had a proportion of CD4^+^CD8^−^HLA‐DR^+^ lymphocytes ≥ 5% of CD4^+^CD8^−^ lymphocytes. However, this single marker was not sufficiently effective to discriminate between patients since 35.8% (54/151) of patients who did not deliver within 7 days met this criterion. Therefore, we chose to combine a second marker and identified CD4^+^CD8^+^HLA‐DR^+^ lymphocytes. Among women with more than 5% of CD4^+^CD8^−^HLA‐DR^+^ lymphocytes, those who delivered within 7 days had a proportion of CD4^+^CD8^+^HLA‐DR^+^ lymphocytes ≥ 20% of CD4^+^CD8^+^ lymphocytes (Figure 3). Thus, the combination of these two consecutive markers allowed us to identify 57% (95/167) of women hospitalized for PTL with a zero probability of delivering within 7 days while retaining all the patients who delivered within 7 days post admission.

Among the suspected causes of PTL and delivery, infection and/or inflammation is a pathologic process for which both a strong causal relationship with preterm delivery and a molecular pathophysiology have been established [33]. During delivery, the myometrium, cervix, and fetal membranes are massively infiltrated by circulating leukocytes [16, 17] secreting pro‐inflammatory cytokines [18, 19, 20]. Yuan et al. [16] showed that the expression of T cell activation markers (CD26 and CD62L) was higher in laboring women, and the authors suggested that peripheral blood T cells were primed for activation during labor. This observation is in line with our results. Indeed, the activation status of CD4^+^CD8^−^HLA‐DR^+^, CD4^−^CD8^+^HLA‐DR^+^, and CD4^+^CD8^+^HLA‐DR^+^ lymphocytes increased dramatically in women who delivered within 7 days compared to those who did not. Consistent with our findings, Zhang et al. [34] found no significant difference in lymphocyte count between PTL and preterm not in labor (PTNIL) women matched for gestational age. Moreover, they described that a significant increase in the activation status of CD3^+^, CD4^+^ T lymphocytes was observed in relation to labor status, indicating their activation during term labor compared to term not in labor (TNIL) and PTNIL [34].

Omics approaches are growing interest in the context of pregnancy and labor [35, 36, 37, 38, 39]. Gomez‐Lopez et al. [35] demonstrated labor‐specific changes in the maternal blood transcriptome, marked by an enrichment of T cell signature, which is consistent with our findings. Metabolomic studies are also a promising area of research that could provide relevant biomarkers to predict PTL [38, 39]. Recently, a lysolipid, 1‐palmitoleoyl‐GPE (16:1)*, was identified as a novel predictor of sPTL [38]. Nevertheless, the omics approach remains a research tool and may not be readily applicable in an emergency context, such as in cases of suspected sPTL. In contrast, our marker‐based approach, subject to validation, could be implemented almost immediately in most maternity hospitals in countries with a level of care at least equivalent to that in France.

Herein, we demonstrate that activated double‐positive CD4^+^CD8^+^ T cells might predict delivery in the context of sPTL. This rare population has been little studied in the field of pregnancy and labor. However, increased levels of these cells have been observed in different autoimmune diseases such as multiple sclerosis, and rheumatoid arthritis and is considered as a marker for severity in several inflammatory diseases. Their function remains controversial, with conflicting reports describing cytotoxic or suppressive roles for these cells [40, 41]. Therefore, further studies are needed to decipher their role in labor‐associated mechanisms.

It can be argued that other markers exist to assess the risk of PTB, such as fetal fibronectin measurement, combined or not with cervical length measurement. Numerous studies evaluating the diagnostic value of combining transvaginal cervical length measurement with fetal fibronectin testing to identify women at risk have shown conflicting results [42, 43, 44]. Furthermore, despite numerous publications, these markers have not entered routine clinical practice because they did not meet the expectations of obstetricians, namely a 100% NPV for PTB. For example, one study showed that while quantitative fetal fibronectin assays in women with a cervix <25 mm showed an increasing likelihood of preterm delivery with increasing concentrations and a threshold at 200 ng/mL, almost 10% of women with a fetal fibronectin <10 ng/mL delivered preterm [42]. Additionally, it is well known that agreement and reliability of cervical length measurements differed substantially between examiners. If cervical length measurements are used to guide management there is potential for both over‐ and under‐treatment [43]. Our goal was to develop an easily accessible marker to rule out the onset of labor in patients with threatened PTL. Our markers are not intended to replace clinical or imaging parameters but could be combined with existing markers to create a composite score.

In our study population of women admitted for PTL, a tiny minority gave birth within 7 days, and a majority had a normal or near‐normal term (38 ± 2.8 WOG). The use of lymphocyte immunophenotyping allowed us to identify, retrospectively, 57% of women who we could be certain would not give birth within 7 days. Prediction of sPTB by immunophenotyping would allow clinicians to reassure low‐risk women and ensure their management as part of routine antenatal care. It could also have a positive impact on healthcare organization, and health expenditure as unnecessary hospitalization of these pregnant women is associated with a significant socioeconomic burden [45]. Prenatal administration of corticosteroids increases maternal leukocyte and fasting blood glucose levels, and after multiple courses, pregnant women are at risk of infection [46]. This study could also limit the neonatal sequelae associated with exposure to multiple courses of pulmonary maturation glucocorticoids during the prenatal period [47]. Corticosteroid therapy can cause long‐term sequelae in children, such as neurodevelopmental, cardiometabolic, and inflammatory disorders [46].

It is important to emphasize that the technique used for lymphocyte phenotyping is the one used by routine biological laboratories to provide blood count results and that the markers we have evaluated are surface markers that do not require membrane permeabilization procedures. If the clinical interest of our markers is validated, their use could be foreseen in all laboratories performing routine multicolor flow cytometry.

The first strength of our study is that we consecutively recruited 167 women hospitalized for PTL in two tertiary care centers, based on the clinicians’ opinion and centers’ protocols, which therefore reflected the clinical situation in daily practice. Our study is based on a marker that would require minor adaptation to be translated into clinical practice and therefore could easily guide the management of women presenting for PTL. Second, we took a pragmatic clinician's approach by applying the following reasoning: if we want to limit unnecessary hospitalizations, it seems to us less dangerous to wrongly hospitalize a woman who will not give birth within 7 days than to discharge, or fail to transfer to an appropriate care center, a woman who will give birth to a premature baby within 7 days. This is what guided our choice of biomarker thresholds and prioritization of sensitivity and NPV.

We recruited women from two tertiary centers belonging to the same region, that is, with a similar patient typology and PTL management approach. This obviously calls into question the applicability of our results to women from different geographical and genetic backgrounds.

Furthermore, the number of women recruited, although significant, was limited, which also limits the representativeness of our sample in relation to the French population. While our patients were enrolled prospectively, the biomarker analysis was performed retrospectively, after the women's pregnancy outcome was known. We now need to perform a validation cohort, that is, an analysis of markers at the time of women's arrival, with prospective identification of those who are considered to have a zero probability of giving birth within 7 days. Of course, for this validation cohort, management will remain as decided by the clinicians and will not be modified by our classification algorithm. Ideally, this prospective validation cohort should compare the accuracy of lymphocyte phenotyping with that of the combination of fetal fibronectin with cervical length measurement.

To further improve our prediction model, combining pro‐inflammatory cytokine levels with T cells phenotype would be of particular interest. In a recent paper, Svenvik et al. [48] reported that plasma levels of CXCL8, GM‐CSF, and IL‐6 were higher in women who delivered within 48 h of hospital admission for PTL. Exploring STAT3 and STAT5 signaling pathways in T lymphocytes also appears to be an interesting issue. Indeed, the transition to active labor was marked by immune activation of pro‐inflammatory signaling responses, including STAT3 and STAT5 signaling in CD4 and CD8 T cell subsets [22].

## 5 Conclusions

In conclusion, we have been able to confirm the potential interest of new specific lymphocyte markers which have an excellent NPV for PTB and, as such, could significantly limit unnecessary hospitalizations and their harmful consequences for women and their families. Further studies are needed to prospectively validate this marker and to study the conditions necessary for its use in real life, taking into account the diversity of equipment and human resources available in obstetric departments and biological laboratories.

## Ethics Statement

This study was approved by the French Medicines Agency and the local ethics committee (CPP‐Est, Dijon, France).

## Conflicts of Interest

The authors declare no conflicts of interest.

## Supporting information



Supporting Information

Supporting Information

Supporting Information

Supporting Information

Supporting Information

Supporting Information

Supporting Information

Supporting Information

## Data Availability

The data that support the findings of this study are available from the corresponding author upon reasonable request.
