# Supplementary material for: Immunophenotyping and Activation Status of Maternal Lymphocytes to Predict Spontaneous Preterm Birth in Women With Threatened Preterm Labor: A Prospective Observational Study
Source: Am J Reprod Immunol. 2024 Dec 3;92(6):e70015. doi: 10.1111/aji.70015 (PMC11613301; doi:10.1111/aji.70015)
Supplement: Supplementary file 1 — Supporting Information [file AJI-92-e70015-s003.docx]

**Figure S1:** **Gating strategy for CD4^+^ and CD8^+^ lymphocyte subsets, activation and regulatory cells analysis.** (A) Lymphocytes were primary gated on a CD45/SSC dotplot. (B) CD3^+^ lymphocytes were further gated from the lymphocyte gate using a CD3 histogram. (C) CD4^+^ and CD8^+^ lymphocytes subclasses were further gated from the CD3^+^ gate using a CD4/CD8 dotplot. CD4^+^CD8^-^ cells were identified as CD4^+^ lymphocytes, CD4^-^CD8^+^ cells were identified as CD8^+^ lymphocytes, CD4^+^CD8^+^ cells were identified as Dual Positive lymphocytes and CD4^-^CD8^-^ cells were identified as Gamma Delta lymphocytes. (D) HLA-DR^+^ lymphocytes cells were identified from CD4^+^ and CD8^+^ gates. (E) Regulatory lymphocytes were gated using a CD25/CD127 dotplot. Regulatory CD4^+^ and CD8^+^ lymphocytes were identified as CD25^hi^CD127^lo^ cells in their respective gates.

**Figure S2:** **Gating strategy for NK, NKT and B lymphocytes analysis.** (A) Lymphocytes were primary gated on a CD45/SSC dotplot. (B) Natural Killer (NK) lymphocytes subclasses were further gated from the lymphocytes gate using a CD3/CD16-CD56 dotplot. CD3^-^CD16^+^CD56^+^ cells were identified as NK lymphocytes, CD3^+^CD16^+^CD56^+^ cells were identified as NK T lymphocytes (NKT). (C) B lymphocytes were further gated from the lymphocytes gate using a CD3/CD19 dotplot. CD3^-^CD19^+^ cells were identified as B lymphocytes. (D) HLA-DR^+^ lymphocytes cells were identified from their respective NK, NKT and B lymphocyte gates.

**Figure S3: Gating strategy for T_H_1, T_H_2 and T_H_17 lymphocytes analysis.** Human PBMC were stimulated 4 hours in presence or absence of PMA/ionomycin. (A) Lymphocytes were primary gated on a FSC/SSC dotplot. (B) CD4^+^ Lymphocytes were further gated from the lymphocytes gate using a CD4 histogram. (C) T**_H_**1, (D) T**_H_**2 and (E) T**_H_**17 lymphocytes were further gated from the CD4^+^ population using a INFꝩ, a IL-4 and a IL-17 histogram respectively.

**Figure S4: CD4^+^ and CD8^+^ lymphocyte populations and activation status according to delivery or not within 7 days post admission.** Percentages of lymphocytes are represented as scatter dot plot while Median Fluorescence Intensity of labelled cells is shown as histograms. Percentage of (A) CD4**^+^**CD8**^-^**, (C) CD4**^-^**CD8**^+^**, (E) CD4**^+^**CD8**^+^** lymphocytes; MFI of (B) CD4**^+^**CD8**^-^**HLA-DR^+^, (D) CD4**^-^**CD8**^+^**HLA-DR^+^, (F) CD4**^+^**CD8**^+^**HLA-DR^+^ lymphocytes; (D) Percentage of CD4**^+^** and CD8**^+^** regulatory lymphocytes. *MFI*, median fluorescence intensity; *NS*, non significant.

**Figure S5: Gamma Delta, NK, NKT and B lymphocyte subpopulations and activation status according to delivery or not within 7 days post admission.** Percentages of lymphocytes are represented as scatter dot plot. Percentage of (A) Gamma Delta (B) HLA-DR^+^ Gamma Delta, (C) NK, (D) HLA-DR^+^ NK (E) NKT, (F) HLA-DR^+^ NKT, (G) B and (H) HLA-DR^+^ B lymphocytes. *NS*, non significant.

**Figure S6: T_H_1, T_H_2 and T_H_17 lymphocyte subpopulations according to delivery or not within 7 days post admission.** Percentages of lymphocytes are represented as scatter dot plot (left panels) and MFI of labelled cells is shown as histograms (right panels). Percentage of (A) T**_H_**1, (C) T**_H_**2 and (E) T**_H_**17 lymphocytes. MFI of (B) T**_H_**1, (D) T**_H_**2 and (F) T**_H_**17 lymphocytes. *MFI*, Median Fluorescence Intensity; *NS*, non significant.
