# Supplementary figures and images for: Immunophenotyping and Activation Status of Maternal Lymphocytes to Predict Spontaneous Preterm Birth in Women With Threatened Preterm Labor: A Prospective Observational Study
Source: Am J Reprod Immunol. 2024 Dec 3;92(6):e70015. doi: 10.1111/aji.70015 (PMC11613301; doi:10.1111/aji.70015)

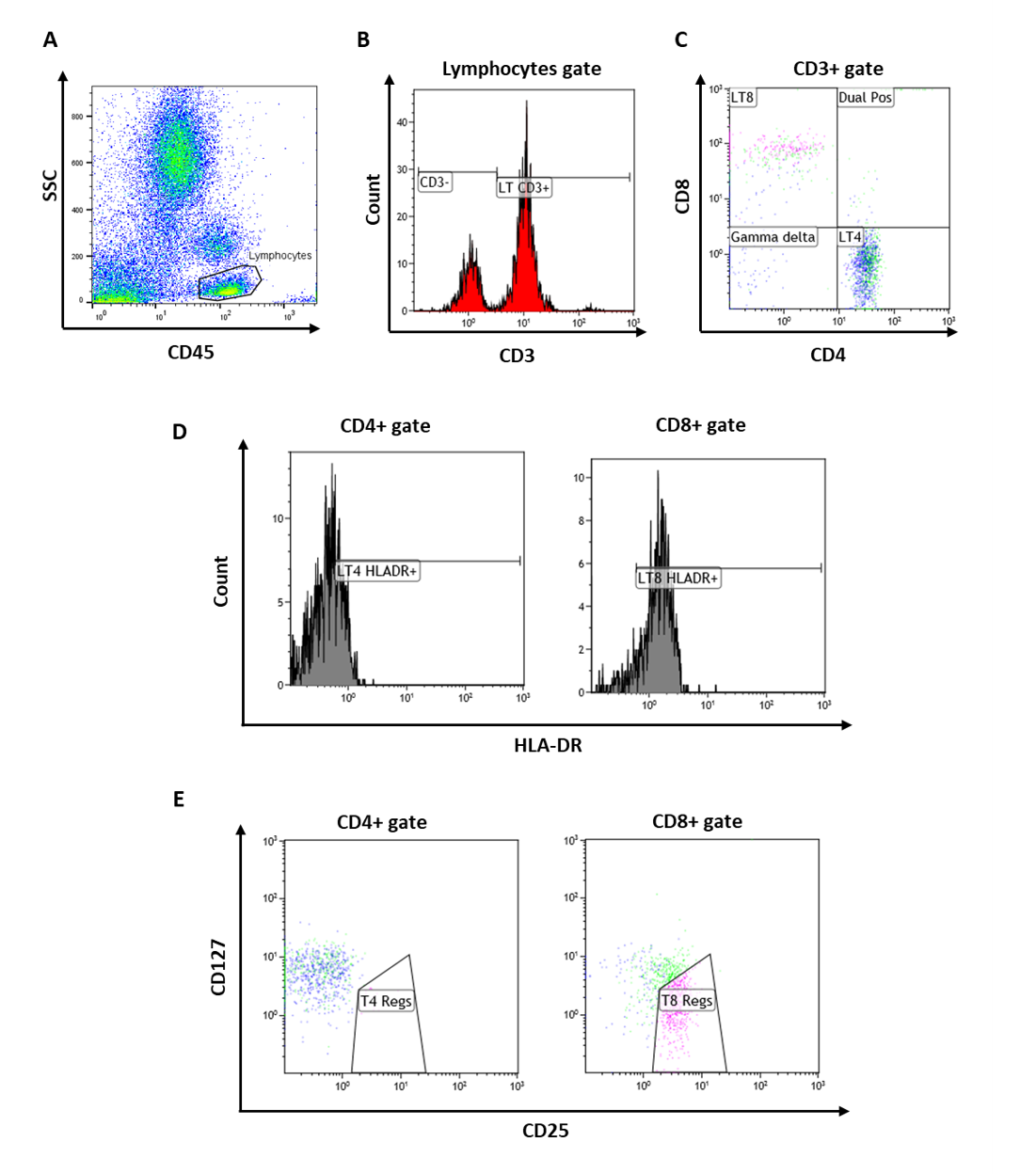

Supplement: Supplementary file 2 — Supporting Information [file AJI-92-e70015-s006.png]

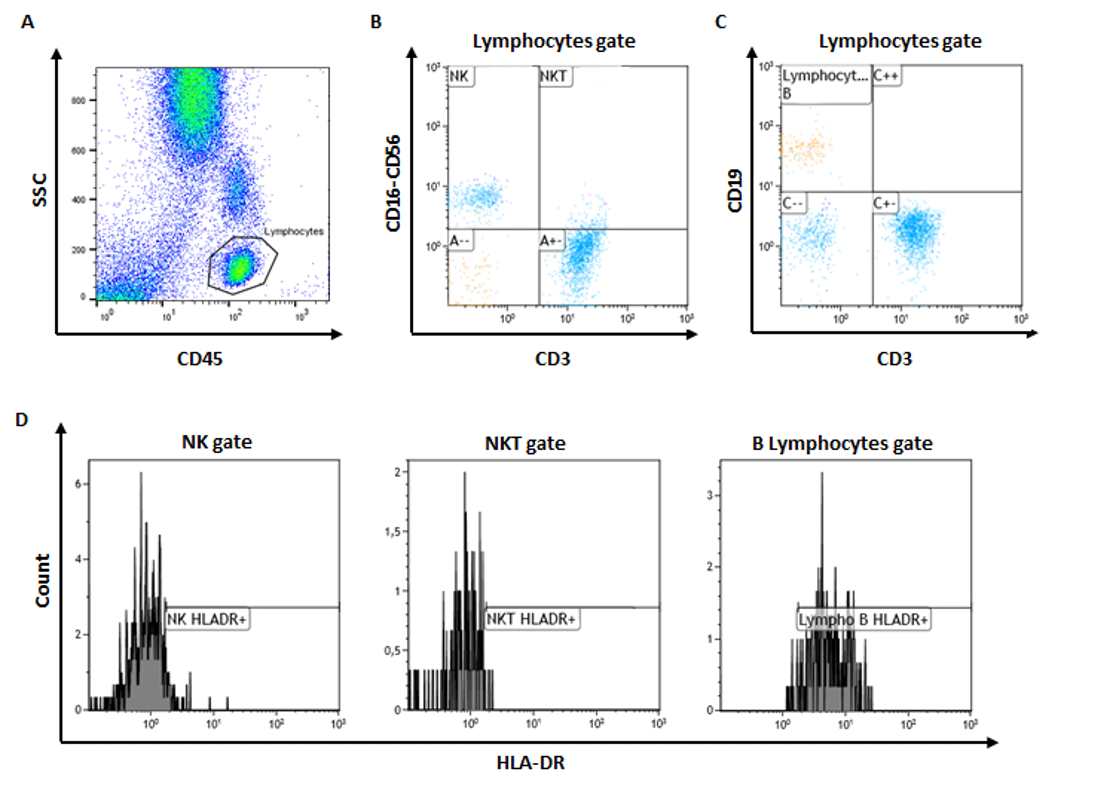

Supplement: Supplementary file 3 — Supporting Information [file AJI-92-e70015-s004.png]

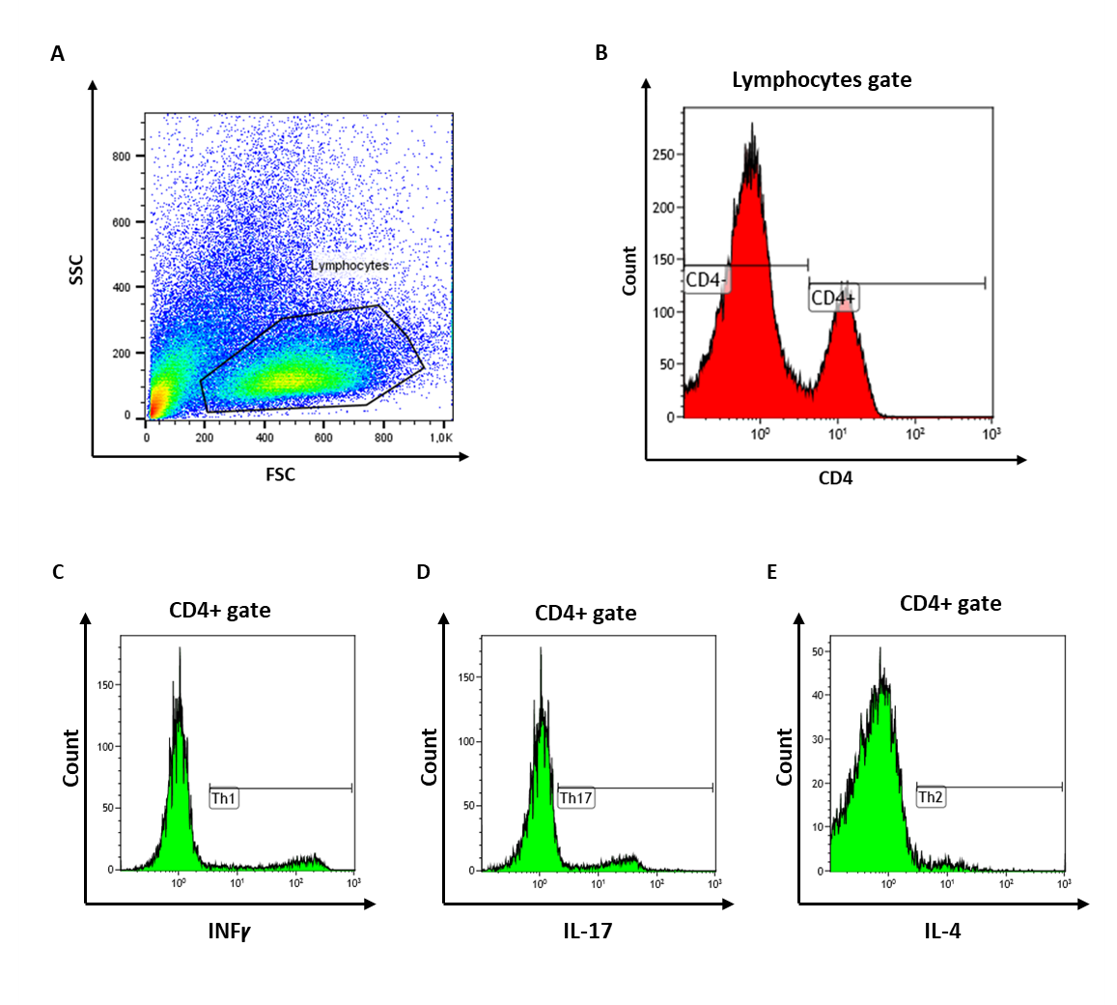

Supplement: Supplementary file 4 — Supporting Information [file AJI-92-e70015-s007.png]

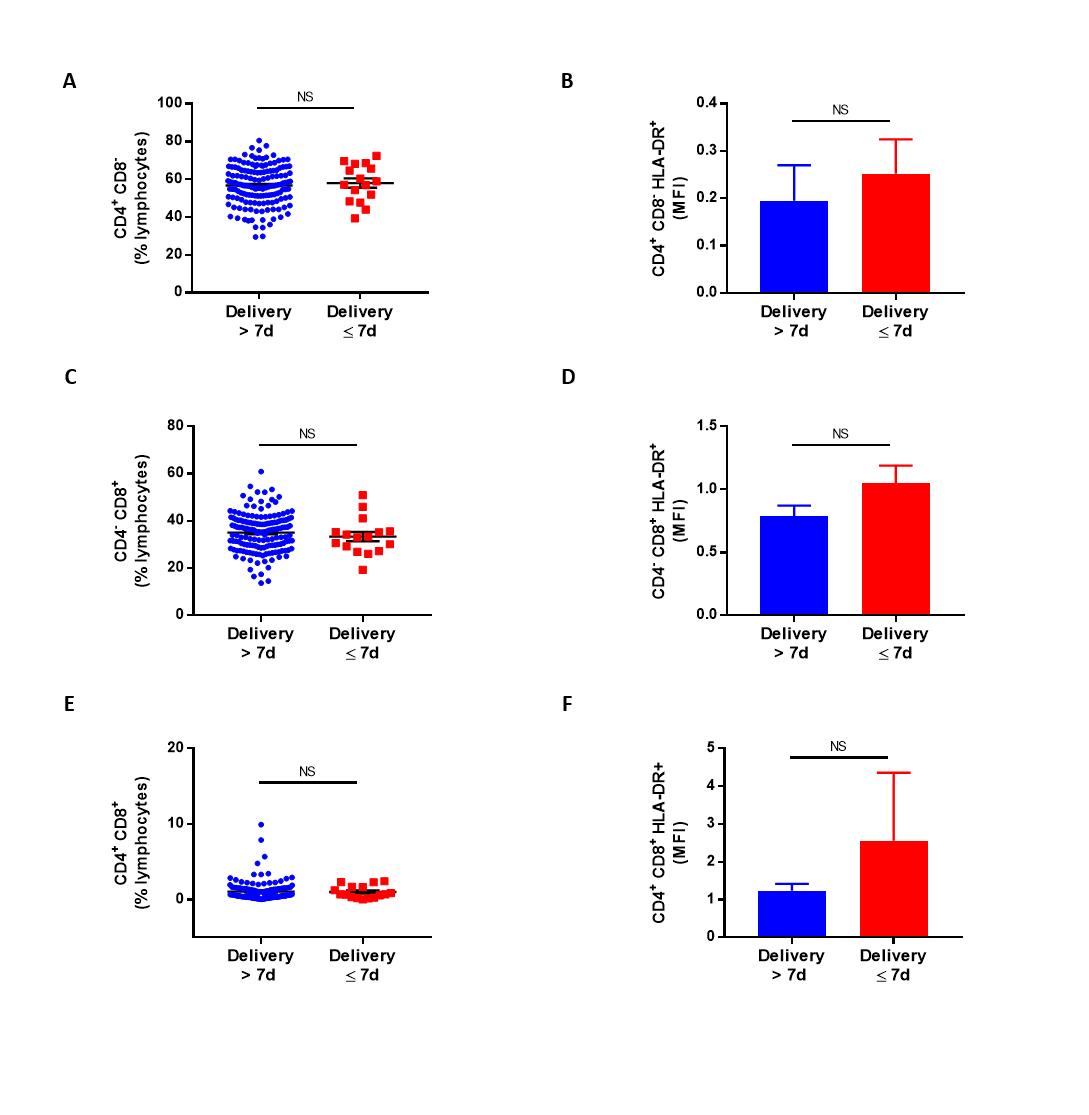

Supplement: Supplementary file 5 — Supporting Information [file AJI-92-e70015-s001.png]

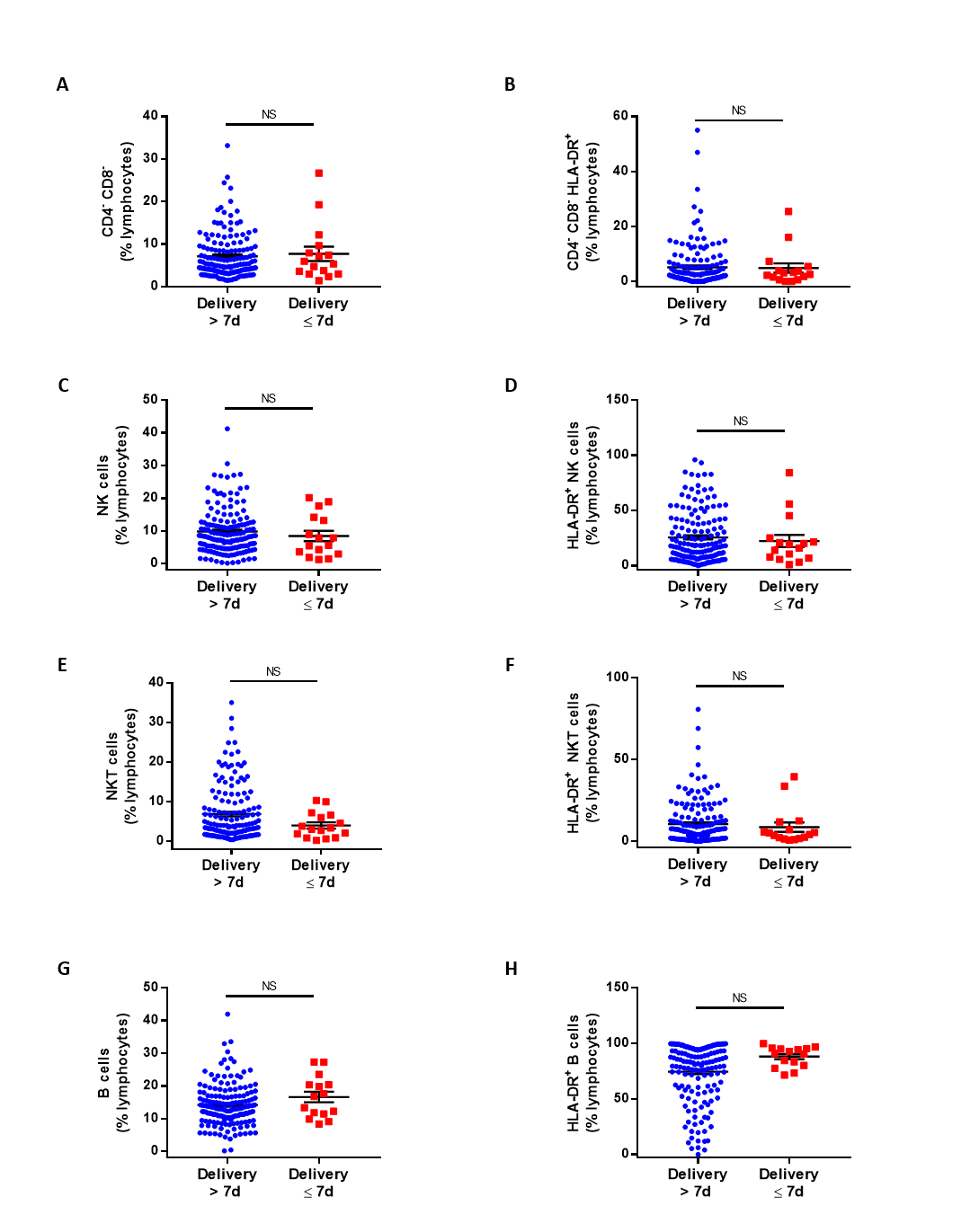

Supplement: Supplementary file 6 — Supporting Information [file AJI-92-e70015-s008.png]

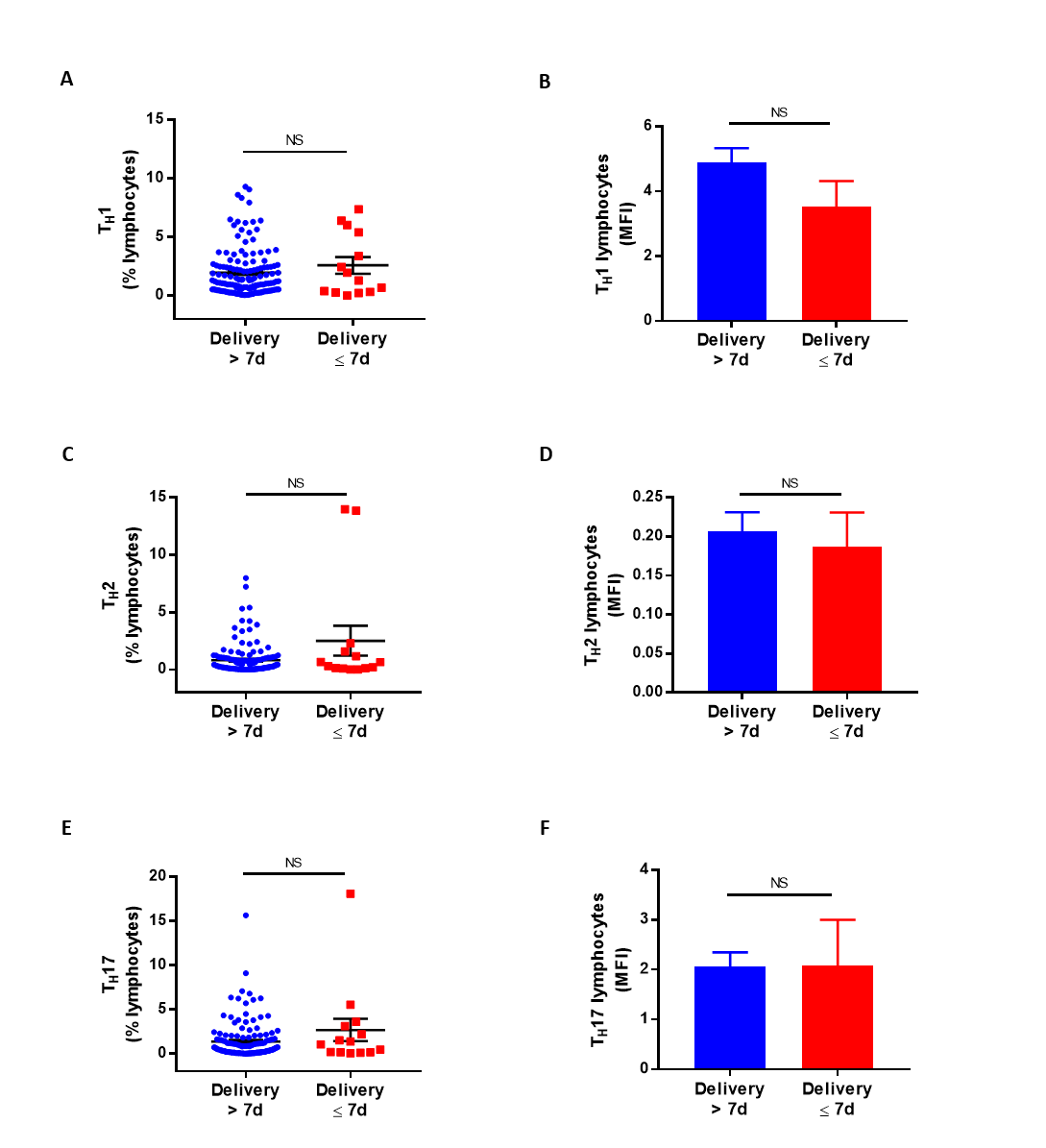

Supplement: Supplementary file 7 — Supporting Information [file AJI-92-e70015-s005.png]
