# Supplementary material for: Immunophenotyping and Activation Status of Maternal Lymphocytes to Predict Spontaneous Preterm Birth in Women With Threatened Preterm Labor: A Prospective Observational Study
Source: Am J Reprod Immunol. 2024 Dec 3;92(6):e70015. doi: 10.1111/aji.70015 (PMC11613301; doi:10.1111/aji.70015)
Supplement: Supplementary file 8 — Supporting Information [file AJI-92-e70015-s002.docx]

**Table S1: Antibodies and fluorophores used for cytometric analyses**

| **Lymphocyte subpopulations** | **Cell marker** | **Fluorophore** | **Reference** |
| --- | --- | --- | --- |
| CD4, CD8 and regulatory T lymphocytes | CD45 | V500 | 560777 |
|  | CD3 | FITC | 555916 |
|  | CD4 | PE-Cy7 | 557852 |
|  | CD8 | PerCP-Cy5.5 | 560662 |
|  | CD25 | APC | 555434 |
|  | CD127 | PE | 557938 |
|  | HLA-DR | APC-H7 | 561358 |
| Natural killer and B lymphocytes | CD45 | V500 | 560777 |
|  | CD3 | FITC | 555916 |
|  | CD16 | APC-Cy7 | 557758 |
|  | CD56 | PE | 555516 |
|  | CD19 | APC | 555415 |
|  | HLA-DR | PE-Cy5 | 551375 |
| T_H_1/T_H_2/T_H_17 lymphocytes | CD4 | PerCP-Cy5.5 | 560751 |
|  | IFN-γ | FITC |  |
|  | IL-4 | APC |  |
|  | IL-17 | PE |  |

**Table S2: Lymphocyte populations ± SEM according to delivery**

| **Lymphocyte population** | **Lymphocyte marker** | **Delivery**  **> 7d** | **Delivery**  **≤ 7d** | ***p*-value^a^** |
| --- | --- | --- | --- | --- |
| CD4^+^ T  Lymphocytes | % CD4^+^CD8^-^ | 56.7 (± 0.8) | 58.0 (± 2.5) | .63 |
|  | % CD4^+^CD8^-^HLA-DR^+^ | 4.8 (± 0.4) | 21.2 (± 2.6) | <.0001 |
|  | MFI CD4^+^CD8^-^HLA-DR^+^ | 0.19 (± 0.07) | 0.25 (± 0.07) | .17 |
|  | % CD4^+^CD8^-^CD25^hi^CD127^lo^ | 2.9 (± 0.2) | 1.4 (± 0.2) | <.0001 |
| CD8^+^ T  Lymphocytes | % CD4^-^CD8^+^ | 35.1 (± 0.7) | 33.4 (± 1.9) | .43 |
|  | % CD4^-^CD8^+^HLA-DR^+^ | 36.0 (± 2.1) | 68.5 (± 6.5) | <.0001 |
|  | MFI CD4^-^CD8^+^HLA-DR^+^ | 0.79 (± 0.08) | 1.04 (± 0.14) | .09 |
|  | % CD4^-^CD8^+^CD25^hi^CD127^lo^ | 52.8 (± 1.9) | 32.0 (± 6.3) | .0007 |
| Dual Positive  Lymphocytes | % CD4^+^CD8^+^ | 1.3 (± 0.3) | 1.0 (± 0.3) | .85 |
|  | % CD4^+^CD8^+^HLA-DR^+^ | 37.2 (± 2.4) | 58.9 (± 6.2) | .0046 |
|  | MFI CD4^+^CD8^+^HLA-DR^+^ | 1.2 (± 0.2) | 2.6 (± 1.8) | .48 |
| Gamma Delta  Lymphocytes | % CD4^-^CD8^-^ | 7.2 (± 0.4) | 7.7 (± 1.7) | .98 |
|  | % CD4^-^CD8^-^HLA-DR^+^ | 5.1 (± 0.6) | 4.9 (± 1.7) | .72 |
| B Lymphocytes | % CD3^-^CD19^+^ | 14.2 (± 0.5) | 16.6 (± 1.6) | .17 |
|  | % CD3^-^CD19^+^HLA-DR^+^ | 74.6 (± 2.1) | 88.1 (± 2.3) | .21 |
| Natural Killer Lymphocytes | % CD3^-^CD16^+^CD56^+^ | 9.8 (± 0.6) | 8.4 (± 1.6) | .42 |
|  | % CD3^-^CD16^+^CD56^+^HLA-DR^+^ | 25.7 (± 1.9) | 22.4 (± 5.5) | .73 |
| Natural Killer T Lymphocytes | % CD3^+^CD16^+^CD56^+^ | 6.9 (± 0.6) | 4.0 (± 0.8) | .19 |
|  | % CD3^+^CD16^+^CD56^+^HLA-DR^+^ | 10.5 (± 1.1) | 8.6 (± 2.9) | .78 |
| T Helper  Lymphocytes | % CD4^+^IFN-γ^+^ (T_H_1) | 2.1 (± 0.2) | 2.6 (± 0.7) | .81 |
|  | MFI CD4^+^IFN-γ^+^ (T_H_1) | 4.9 (± 0.5) | 3.5 (± 0.8) | .49 |
|  | % CD4^+^IL-4^+^ (T_H_2) | 0.8 (± 0.1) | 2.5 (± 1.3) | .51 |
|  | MFI CD4^+^IL-4^+^ (T_H_2) | 0.21 (± 0.02) | 0.19 (± 0.04) | .62 |
|  | % CD4^+^IL-17^+^ (T_H_17) | 1.4 (± 0.2) | 2.7 (± 1.3) | .37 |
|  | MFI CD4^+^IL-17^+^ (T_H_17) | 2.1 (± 0.3) | 2.1 (± 0.9) | .95 |

*SEM, standard error to the mean; MFI, median fluorescence intensity*

^a^ p-values estimated using t test to compare the two groups.
